# Assessing the environmental impacts of EU consumption at macro-scale

**DOI:** 10.1016/j.jclepro.2019.01.134

**Published:** 2019-04-10

**Authors:** Antoine Beylot, Michela Secchi, Alessandro Cerutti, Stefano Merciai, Jannick Schmidt, Serenella Sala

**Affiliations:** a European Commission, Joint Research Centre, Via Enrico Fermi 2749, I-21027 Ispra, VA, Italy; b 2.-0 LCA Consultants, Rendsburggade 14, room 1.431, 9000, Aalborg, Denmark; c Danish Center for Environmental Assessment (DCEA), Department of Planning, Aalborg University, Rendsburggade 14, room 1.431, 9000, Aalborg, Denmark

**Keywords:** Impact assessment, Consumption, Input-output analysis, EXIOBASE 3, LCIA

## Abstract

Sustainable Consumption and Production is one of the leading principle towards reducing environmental impacts globally. This study aims at combining Environmentally-Extended Input-Output Analysis (using EXIOBASE 3) with up-to-date impact assessment models to quantify the environmental impacts induced by final consumption in the EU Member States in 2011. The environmental extensions are characterized in 14 environmental impact categories out of the 16 used in the Environmental Footprint life cycle impact assessment method. A contribution analysis of key products and services as well as emissions and resources, which drive the environmental impacts of EU consumption, is conducted. Environmental impacts are mainly induced along the supply-chain of products and services. Several expenditures relative to services represent large shares both in the total final consumption and in the 14 impacts under study, despite a relatively low impact intensity. Food products, in particular meat and dairy products, are identified as key contributors regarding acidification, eutrophication, land use, and water use, and to a lower extent climate change. Finally, several manufactured products, raw materials and basic products respectively importantly contribute to impacts on human toxicity, freshwater ecotoxicity and resource uses. The total volume of final consumption expenditures per EU Member State appears a key explanatory variable to most of the impacts embodied in their consumption, yet to a lower extent regarding water use and fossils resource use. Finally, the current limitations in using EXIOBASE 3 for environmental impact assessment are discussed, with specific attention to EXIOBASE environmental extensions and to the case study on EU consumption. Since the classification of emissions and resources for impact assessment requires a number of assumptions that may influence the results, a sensitivity analysis is performed to exemplify some of the key issues relative to the characterization of impacts based on EXIOBASE environmental extensions.

## Highlights

• Environmental impacts induced by final consumption in EU28 in 2011 are assessed.
• Environmental extensions are characterized in 14 environmental impact categories following the Environmental Footprint method.
• Contribution of key products and services and key emissions and resources is analyzed.
• The total volume of consumption per EU Member State is a key explanatory variable.
• Current limitations in using EXIOBASE 3 for environmental impact assessment are discussed.

## 1 Introduction

The consumption and production of products (goods and services) are responsible for adverse effects to the environment, encompassing effects on human health and natural resources. Ensuring sustainable consumption and production patterns is the goal 12 of the UN Agenda 2030 (UN, 2015), and it has been considered an overarching objective and an essential requirement for sustainable development (UN, 2012). In the European Union, Sustainable Consumption and Production (SCP) is targeted through a number of policy instruments, as subsumed under the Sustainable Consumption and Production Action Plan (European Commission, 2008) and the Circular Economy Action Plan (European Commission, 2015). These are intended to “improve the overall environmental performance of products throughout their life cycle, stimulate demand for better products and production technologies, and help consumers make informed choices” (European Parliament, 2017). Moreover, in the 7th Environmental Action Program (EAP) (EU, 2013), the European Union (EU) has a long term objective of living well within the planet's ecological limits, implying a significant decoupling of environmental impact from economic growth and welfare. This is pivotal as well in the context of the Beyond GPD discussion, namely measuring progress, true wealth, and well-being (European Commission, 2009). Despite the relevance of this decoupling, assessing the environmental sustainability of production and consumption is challenging and, nowadays, mainly performed via the assessment of pressure indicators of emissions and resource consumption, barely addressing potential impacts to environment and human health. This is the case as well of the indicators selected, so far, for assessing the sustainable development goal (SDG) 12 both at United Nations (UN, 2017) and EU level (EU, 2018).

At the level of national economies, environmental accounting distinguishes two ways to quantify emissions to the environment and primary resources consumption. On the one hand, the producing economic agent is considered responsible for the emissions and consumptions directly resulting from the production of goods and services. This “production perspective” is implemented to calculate the “domestic footprint” of nations, based on domestic inventories of emissions and resource extraction (see e.g. Benini et al., 2014). On the other hand, in the second (“consumer”) perspective, the final demand for goods and services is considered to induce emissions to the environment and resources extraction along these goods and services' supply-chain, in addition to direct emissions generated by the final use of goods (Munksgaard and Pedersen, 2001).

This second perspective has been increasingly used in the last decades to calculate the environmental footprint of consumption, from Leontief's works laying the foundations of the input-output approach (Leontief, 1970) to the recent developments of Environmentally-Extended Multi-Regional Input-Output (EE-MRIO) databases, including in particular EXIOBASE versions 2 and 3 (Wood et al., 2015; Stadler et al., 2018; Merciai and Schmidt, 2018), WIOD (Dietzenbacher et al., 2013; Timmer et al., 2015) and EORA (Lenzen et al., 2012, 2013a). EE-MRIO databases tend to render obsolete the long-made “domestic technology assumption”, which consists in assuming that the imported products are produced using the same technology as in the country under study. Moreover, these databases have been built generally considering a larger level of disaggregation of products and services, as well as a larger set of environmental extensions.

EE-MRIO databases have recently been implemented in a number of studies claiming to address the “environmental footprint” or the “environmental impacts” of nations. However, the current existing literature is still essentially limited to a focus on either carbon (Boitier, 2012; Moran and Wood, 2014; Ivanova et al., 2016, 2017; Sodersten et al., 2018; Schmidt and Muños, 2014), material (Pothen and Schymura, 2015; Wiedmann et al., 2013), land or water footprints (Lenzen et al., 2013b), with some studies considering the four of them (Tukker et al., 2016; Wood et al., 2018). Beyond these four flows, which have represented the main focus so far, other flows such as waste (Tisserant et al., 2017) and nitrogen (Oita et al., 2016) have been additionally scrutinized. Therefore, up to now, existing studies have primarily limited their analysis to a reduced set of flows, in most cases without any quantification of the corresponding impacts these flows induce on the environment. Only recently, Steinmann et al. (2018) have used EXIOBASE 3 to quantify the potential impacts generated by the pressures (that is, by the flows) induced by consumption, and subsequently to identify a limited set of environmental indicators that explain most of the variance of the total impacts embedded in EXIOBASE 3. Similarly, Hamilton et al. (2018) have considered two impact categories, marine and freshwater eutrophication, to characterize the importance of overall consumption in the world over the period 2000–2011 by use of EXIOBASE 3.

In this context, this work combines EXIOBASE 3 with up-to-date impact assessment models to quantify the environmental impacts induced by final consumption in the 28 countries of the EU. The aim is to assess to which extent the application of impact assessment models may support the assessment of potential environmental impact beyond the mere pressure-based footprints. A key aspect of this research relies in the mapping between the inventory of elementary flows as calculated from the application of EXIOBASE 3, and the corresponding characterization factors available in up-to-date impact assessment methods, which enable to calculate the potential impacts these flows induce on the environment. From the impact assessment results, a contribution analysis of products and services and of substances driving the environmental impacts of EU consumption is conducted. However, to comprehensively assess the potential impacts of consumption, and the decoupling between environmental impacts and economic growth, there are still limitations that should be systematically presented and addressed in future development. Hence, the current limitations in using EXIOBASE 3 for environmental impact assessment are discussed, with specific attention to EXIOBASE environmental extensions and to the case study on EU consumption. The sensitivity of the impact assessment results to the mapping of elementary flows is specifically discussed.

## 2 Method

This section introduces firstly the standard input-output approach, and secondly the EXIOBASE 3 database implemented to calculate the pressures induced by EU consumption. Finally, the last sub-section describes the procedure undertaken to assign characterization factors (as available in up-to-date midpoint impact assessment methods) to the inventory of elementary flows derived from EXIOBASE 3, and to calculate the corresponding potential environmental impacts. As a complement, the Supporting Information (SI) (1) provides a practical description of the approach undertaken to perform the calculations (including e.g. where to find the hybrid version of EXIOBASE 3, which files to use, etc.), in order to ease the implementation of similar studies in the future.

### 2.1 Multi-regional input-output analysis

The standard economic input-output model is based on the economic identity between the total output of an economic sector and the sum of the demand for that sector's output from other sectors plus the final demand. Considering x the vector of output productions, I the identity matrix, A the technological requirement matrix and f the final demand, the Leontief inverse equation is derived according to:

(1) x=Ax+f

(2) $$x=\;{\left(I-A\right)}^{-1}f$$

The inventory (g) of emissions to the environment, and of resources extracted from the environment, as a response to a given final demand is then calculated according to:

(3) $$g=Bx=B{\left(I-A\right)}^{-1}f$$

with B the matrix of sectorial resources and emissions intensities, reporting the sectorial coefficients of natural resources extraction and emissions per unit of output in the sector.

MRIO models extend the standard IO matrix to a larger system where each industry in each region has a separate row and column (Hertwich and Peters, 2010). Considering x_i_ the vector of output in region i, A_ij_ the technological requirement matrix from region i to region j, f_ij_ the final demand vector from region i to region j, and N the number of regions differentiated, then equation (1) reads:

(4) $$\left(\begin{array}{l} x_{1}\\ ⋰\\ x_{i}\\ ⋰\\ x_{N} \end{array}\right)=\left(\begin{array}{lllll} A_{11} & \cdots & A_{1j} & \cdots & A_{1N}\\ ⋰ & \ddots & ⋰ & \ddots & ⋰\\ A_{i1} & \cdots & A_{ij} & \cdots & A_{iN}\\ ⋰ & \ddots & ⋰ & \ddots & ⋰\\ A_{N1} & \cdots & A_{Nj} & \cdots & A_{NN} \end{array}\right)\left(\begin{array}{l} x_{1}\\ ⋰\\ x_{i}\\ ⋰\\ x_{N} \end{array}\right)+\sum_{i=1}^{N}\left(\begin{array}{l} f_{1i}\\ ⋰\\ f_{ji}\\ ⋰\\ f_{Ni} \end{array}\right)$$

and equation (3) is modified accordingly.

### 2.2 EXIOBASE 3

This study aims at assessing the environmental impacts embodied in EU consumption, considering the final consumption (that is, the sum of expenditures from households, from government and from Non-Profit Institutions Serving Households) of the 28 EU Member States in the year 2011. All elements in equations (3), (4)) are drawn from the hybrid version of EXIOBASE database version 3.3.8 (Merciai and Schmidt, 2018; Stadler et al., 2018), referred to as EXIOBASE 3 in this study. The nomenclature of sectors in EXIOBASE relies on the NACE (Nomenclature statistique des activités économiques dans la Communauté européenne) nomenclature, with further disaggregation regarding some products (in particular agricultural and food products, energy and waste treatment; for further details, see SI document 9 in Stadler et al., 2018). The vector of final consumption (f in equations (3), (4))) differentiates the 28 EU Member States and 137 products and services. Investments are additionally integrated within the IO tables, based on the approach developed in the project FORWAST (Schmidt et al., 2010). All the results presented hereafter therefore account for investments (usually referred to as “capital goods or “infrastructure” in Life Cycle Assessment) whose contribution is attributed to the final consumption.

In EXIOBASE 3, MRIO Tables (matrices A_ij_ in equation (4)) are available for 43 countries, including the 28 EU countries under focus in this study, plus five rest-of-world regions. The matrices of resources and emissions intensities (B in equation (3)) distinguish 164 sectors with respect to 48 countries and regions, and report coefficients relative to 78 elementary flows: 36 mineral, metal and energy resources, 5 types of land occupation, 3 types of water consumption, and 29 substances emitted to air, 2 to water and 3 to soil.

### 2.3 From elementary flows to impacts

Equation (4) describes the standard calculation implemented in current IO literature, that is most often limited to flows (carbon, material, water or land). When considering further the environmental impacts induced by consumption, the potential impacts generated by pressures need to be characterized. Considering *g* the vector of environmental pressures induced by final consumption in EU28 (of dimension 1 × 78 elementary flows as derived from EXIOBASE 3), the corresponding impacts are calculated according to:

(5) e=Cg

with e the vector of environmental impacts and C the matrix of characterization factors (reporting the impact intensity per unit of resource extracted or substance emitted to the environment).

In this study, 14 impact categories are considered, applying the European Environmental Footprint (EF2017) LCIA method (EC, 2017), namely: climate change; acidification; eutrophication, terrestrial; eutrophication, marine; eutrophication, freshwater; particulate matter; photochemical ozone formation; human toxicity, cancer; human toxicity, non-cancer; ecotoxicity, freshwater; land use; water use; resource use, minerals and metals; and resource use, fossils. Whereas the EF2017 LCIA method also recommends to assess the impacts in terms of ionizing radiation and ozone depletion, these two impact categories are excluded from this study. Indeed, in EXIOBASE 3 (more precisely, in matrix B), coefficients relative to ionizing radiations are missing (as also reported by Huysman et al., 2016) while ozone-depleting substances very often lack a value as well.

The construction of matrix C consisted in assigning a characterization factor (CF) to each of the flows taken into account in the EXIOBASE environmental extensions. This required a systematic classification to the EF2017 nomenclature, briefly explained in the following and more extensively detailed in the Supporting Information (SI document 2). On the one hand, regarding the majority of EXIOBASE elementary flows, especially emissions, a mapping based on a common nomenclature was straightforward. On the other hand, many of the flows reported in EXIOBASE 3 (in EXIOBASE nomenclature) required the implementation of the following strategy for them to be assigned a CF.

Firstly, when it was not possible to match one EXIOBASE extension to a flow in EF2017, a proxy CF was introduced. The selection of the proxy was based on the same pollutants group (e.g. Persistent Organic Pollutants and Persistent Bioaccumulative and Toxic) or on the same chemical group (e.g. dioxins). Secondly, the oxidation state of chromium and arsenic emissions to air is missing in EXIOBASE 3, whereas it very often influences the fate and the effect of the chemical and, subsequently, the impact quantification (regarding human toxicity and ecotoxicity). The “unspecified” CF was therefore assigned to chromium, as available in EF2017, whereas the highest CF was selected regarding arsenic (no “unspecified” CF in that case, but CFs relative to arsenic III and arsenic V). Finally, all the aggregated flows from EXIOBASE needed the calculation of a CF encompassing all the substances included in the aggregation. Each of these CFs was estimated either *i)* as a weighted average considering the amount of emissions or resource produced at EU-27 or at global level, as the preferred solution when feasible, or *ii)* as an arithmetic average of the CFs available in EF2017 for the flows included in the group (see Supporting Information document 2 for further details).

Overall, considering each impact category, a limited number of elementary flows is taken into account for the impact assessment step compared to the total number of flows for which a CF is available in the EF2017 LCIA method (SI document 1). In particular, toxic and ecotoxic impacts are calculated considering 11 to 15 substances, compared to 1321 to 7566 substances characterized in the EF2017 LCIA method. Substances in EXIOBASE 3 assigned with a CF considering human toxicity (cancer and non-cancer) and freshwater ecotoxicity are almost entirely emissions to air, whereas emissions to soil and water represent a share comparable to emissions to air in EF2017. Similarly, non-toxic impacts are calculated with taking into account from 2 to 5 elementary flows, compared to 7 to 212 substances differentiated in the EF2017 LCIA method. Finally, similar observations can be made regarding resource use (e.g. 13 resource flows assigned with a CF in EXIOBASE 3 with respect to minerals and metals resource use, compared to 48 for which a CF is available in EF2017).

This lower number of substances characterized (and therefore contributing to impacts in the assessment) using EXIOBASE 3 compared to the full set of substances available in EF2017 is essentially due to the absence of the corresponding substances in the EXIOBASE environmental extensions. The aggregation of some elementary flows in EXIOBASE (e.g. “other industrial minerals”) also additionally contributes to this discrepancy in the number of substances characterized. On the contrary, six elementary flows calculated from EXIOBASE 3 have been unmapped (that is, these substances have been excluded from the impact characterization) in the absence of the corresponding flows (or proxy flows) in the EF2017 LCIA method.

## 3 Results and discussion

This section firstly describes the total environmental impacts of EU consumption, before analyzing and discussing respectively the contributions of products/services and substances leading the overall impact, and the main contributions of Member States to the total impacts of EU28. In a final sub-section, the current limitations in using EXIOBASE 3 for environmental impact assessment are discussed, with specific attention to EXIOBASE environmental extensions and to the case study on EU consumption.

### 3.1 Total environmental impacts of EU consumption

The environmental impacts induced by European final consumption are assessed considering 14 impact categories (Table 1). In particular, the climate change footprint of EU consumption is evaluated to 6760 million tonnes CO_2_ eq. for the year 2011. Regarding 11 impact categories out of the 14 under study, impacts from economic activities upstream and downstream final consumption (respectively, products and services production and distribution, and solid waste and wastewater treatment) represent more than 87% of the total impacts of EU consumption (Table 1), while direct emissions in the consumption phase (mainly from households) account for the remaining contribution. However, direct emissions in the consumption phase still stand for respectively 78% and 59% of the total impacts in terms of particulate matter and photochemical ozone formation. It is noteworthy that, when considering direct emissions of particulate matter (PM_2.5_) and Non-Methane Volatile Organic Compounds (NMVOC) from final consumption expenditures as available in EXIOBASE 3.3.14 as a substitute to the values from EXIOBASE 3.3.8, a different picture is observed. In that case, direct emissions in the consumption phase stand for respectively 15% and 38% of the total impacts in terms of particulate matter and photochemical ozone formation. Similarly, yet to a lower extent, direct emissions also represent a relatively large contribution (21%) with respect to climate change.

**Table 1:** Environmental impacts of EU final consumption in 2011, considering 14 impact categories of the EF2017 LCIA method.

Impact category	Unit	Total	Share of direct emissions and use	Correlation with the volume of expenditure*
AC	mol H^+^ eq	6.92E+10	11%	0.95
EUTT	mol N eq	2.28E+11	13%	0.96
EUTM	kg N eq	1.66E+10	13%	0.95
EUTF	kg P eq	4.20E+08	0%	0.87
LU	pt	1.10E+15	13%	0.89
WU	m^3^-eq	4.80E+12	4%	0.76
HToxNC	CTUh	6.38E+05	5%	0.90
HToxC	CTUh	3.00E+04	3%	0.87
EcoTox	CTUe	7.25E+11	10%	0.89
PM	disease incidences	3.42E+06	78%	0.39
POF	kg NMVOC-eq	6.98E+10	59%	0.77
CC	kg CO_2_ eq	6.76E+12	21%	0.95
RU-f	MJ	8.47E+13	0%	0.77
RU-mm	kg Sb-eq	3.62E+08	0%	0.83

*Correlation calculated considering the 28 EU Member States (R^2^)

Note on abbreviations of impact categories names: AC: Acidification; EUTT: Eutrophication Terrestrial; EUTM: Eutrophication Marine; EUTF: Eutrophication Freshwater; LU: Land Use; WU: Water Use; HToxNC: Human Toxicity, Non-Cancer; HToxC: Human Toxicity, Cancer; EcoTox: Ecotoxicity freshwater; PM: Particulate Matter; POF: Photochemical Ozone Formation; CC: Climate Change; RU-f: Resource Use, fossils; RU-mm: Resource Use, minerals and metals.

In EXIOBASE, final use is divided into six categories, where household expenditures stand for the largest share in the total final consumption expenditures and at the same time for the largest share in the total environmental impacts of consumption (SI Document 1). Household expenditures have a higher impact intensity compared to other types of expenditures: while representing 69% of the total expenditures, they contribute to [64–76%] of the total impacts regarding five impact categories (with respect to human toxicity, ecotoxicity and resource use), and even up to [81–95%] of the total impacts regarding the nine other impact categories under study. On the contrary, the share of Government expenditures in impacts (between 3 and 24% of the total impacts considering all impact categories) is lower than their share in total expenditures (29%).

### 3.2 Contribution analysis of products/services and substances leading the overall impact

In the modelling, the final consumption of EU28 is divided into 137 categories of products and services, considering the EXIOBASE nomenclature (137 products in use out of the 164 products differentiated in total in the database). On the one hand, a restricted set of “key” products and services is identified on the basis of their “large” contribution to all impact categories (“large” to be understood here as “in comparison with other products and services”). On the other hand, some categories of products and services contribute relatively importantly to one or some impact categories (and are “key” to these impact categories), but in a limited manner to other impact categories.

At this stage, it is to be noted firstly that the analysis is performed considering the EXIOBASE nomenclature for products and services, which mixes a description of products and services on the one hand at the level of divisions according to the NACE, and on the other hand at a more disaggregated level (specific to EXIOBASE). This approach to results analysis enables to take advantage of the relatively large resolution of the EXIOBASE database, but it implies at the same time that some categories of products and services (e.g. Health and social work services) are represented considering a larger level of aggregation compared to other products and services (e.g. food products and electricity). This tends to underestimate the ranking of these disaggregated products and services in the total contribution to impacts. Secondly, it is also to be noted that, in the following, the impacts associated with each product and service category correspond to the sum of direct and indirect (along the supply-chain) impacts. For example, Health and social work services, Public administration and defense services; compulsory social security services, Hotels and restaurants services and Education services include the consumption of food of employees, patients and customers. Therefore, the final consumption of these services bears the impact of this intermediate, inter-sectoral, food consumption.

#### 3.2.1 Contribution analysis of products and services most affecting all impacts

Nine categories of products and services, out of the 137 considered to represent the EU final consumption, appear in the so-called “Top 20” products and services with respect to all impact categories (that is, are one of the 20 most contributing categories of products and services with respect to the 14 impact categories under study; SI document 1). Six out of these nine categories are services: Health and social work services, Public administration and defense services; compulsory social security services, Hotels and restaurants services, Education services, Recreational, cultural and sporting services and Real estate services. These six categories of services represent approximately half of the total volume of EU final consumption expenditures (Eurostat, 2018), but in the meantime have a low impact intensity, in impact units per euro of expenditure, compared to the average impact intensity of EU consumption. For example, expenditures on Health and social work services appear as one of the six categories of products and services most contributing to the environmental impacts of EU consumption, regarding the 14 impact categories under study, while for most impact categories their corresponding impact intensity represents between 36 and 54% of the average impact intensity of EU final consumption (SI document 1). Therefore, the large volume of expenditures relative to some services drives their significant contribution to the total impacts of EU consumption, in a context where these services have a relatively low impact intensity. Finally, beyond the six services mentioned above, three product categories are also part of the Top 20 products and services for the whole list of impact categories under study: Food products not elsewhere classified (nec.), Products of chemicals nec and Motor vehicles, trailers and semi-trailers. These three products are characterized by a lower volume of final consumption compared to services, and in the meantime by a larger impact intensity (SI document 1).

#### 3.2.2 Acidification, eutrophication, land use and water use

**Fig. 1:**
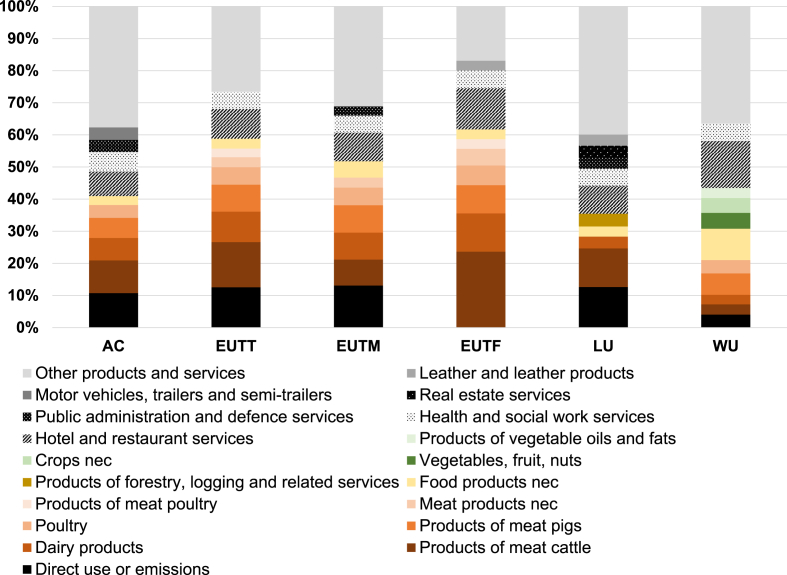
The ten categories of products and services (including direct emissions and use) most contributing to impacts of EU final consumption considering Acidification (AC), Eutrophication Terrestrial (EUTT), Eutrophication Marine (EUTM), Eutrophication Freshwater (EUTF), Land Use (LU) and Water Use (WU).

Now considering the 10 categories of products and services most contributing to impacts (that is, the “Top 10”), a similar set of key products and services is firstly observed with respect to acidification, eutrophication (terrestrial, marine, and freshwater), land use and water use. Out of the 137 categories of products and services of the EU final consumption, a restricted list of 18 categories covers the “Top 10” contributors (including direct emissions) for these six impact categories (Fig. 1). In total, still considering these six impact categories, the “Top 10” categories of products and services (including direct emissions) contribute to more than 60% of the impacts of final consumption (and even up to 83% in the case of freshwater eutrophication).

Meat products and dairy products show a large contribution to impacts on acidification and eutrophication (terrestrial, marine, and freshwater), and to a lower extent on land use and water use, relatively to other products and services (Fig. 1). Products of meat cattle, Products of meat pigs, Poultry, Products of meat poultry, Meat products nec, and Dairy products are part of the Top 10 products and services for most impact categories among acidification, eutrophication, land use and water use. Their summed contributions represent 32%–59% of the total impacts of EU final consumption regarding acidification and eutrophication, and around 16–17% regarding land use and water use. Products of meat cattle even contribute, as a single category, to 24% of the total impacts of EU final consumption on freshwater eutrophication, and to 14% regarding terrestrial eutrophication. Moreover, specifically regarding water use, Vegetables, fruits, nuts, Crops nec, and Products of vegetable oils and fats appear as key products as well, with contributions to impact summing up to 13% of the total. Overall, the consumption of food products, beverages and tobacco products, as re-aggregated according to the NACE nomenclature, contributes to 28% to 57% of the total impacts on acidification, eutrophication (terrestrial, marine, and freshwater), land use and water use, whereas representing only 5% of the total final consumption expenditures (Eurostat, 2018).

Moreover, expenditures relative to several services appear significant regarding these six impact categories. Firstly, expenditures relative to Hotels and restaurants services represent from 8% to 15% of the total impacts. Part of this contribution is induced by the consumption of food products (in particular, of meat) and should be accounted for when analyzing not only the contribution of food products, but instead of the whole food system. Secondly, as previously observed regarding the 14 impact categories under study, the relatively large volumes of expenditures relative to some services (e.g. Health and social work services) imply that these services represent a relatively large share in impacts.

**Fig. 2:**
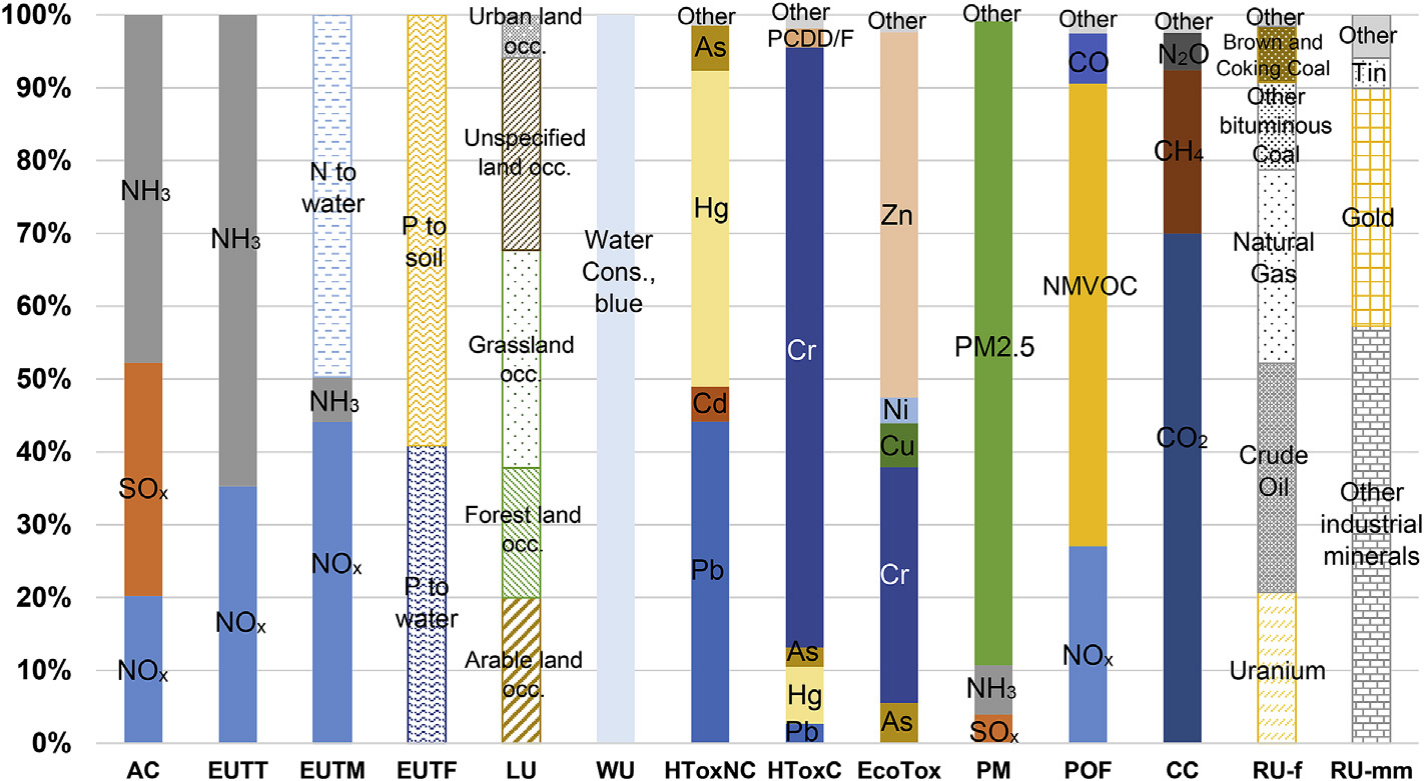
Overview of the main contributing elementary flows, considering 14 impact categories characterized with EF characterization factors. Unless specified, contributing emissions are emissions to air. Elementary flows contributing less than 2.5% are accounted for as “Other”. Note on abbreviations: Occ. stands for Occupation; Cons. stands for Consumption. Impact categories: AC: Acidification; EUTT: Eutrophication Terrestrial; EUTM: Eutrophication Marine; EUTF: Eutrophication Freshwater; LU: Land Use; WU: Water Use; HToxNC: Human Toxicity, Non-Cancer; HToxC: Human Toxicity, Cancer; EcoTox: Ecotoxicity freshwater; PM: Particulate Matter; POF: Photochemical Ozone Formation; CC: Climate Change; RU-f: Resource Use, fossils; RU-mm: Resource Use, minerals and metals.

Additionally, the analysis at the level of elementary flows enables to interpret further the relatively large contribution of products and services, in particular of food products, regarding these six impact categories (Fig. 2). Ammonia (NH_3_) is the most contributing substance to impacts on acidification and terrestrial eutrophication, respectively representing 48% and 65% of the total impacts of EU final consumption. More than half of NH_3_ embodied in EU consumption is emitted along the supply chain of meat and dairy products. Moreover, SO_x_ and NO_x_ emissions to air are the second largest contributors to impacts respectively on acidification (32% of the total impact) and terrestrial eutrophication (35%). NO_x_ emissions to air additionally represent the second largest contribution in terms of marine eutrophication (44%), while emissions of N to water are the most contributing ones (50%). Similarly, the impact on freshwater eutrophication is induced by emissions of respectively P to soil (60%) and P to water (40%). In EXIOBASE 3, emissions of N and P to water and soil are accounted for as generated only by agricultural and waste treatment activities, resulting in the key contribution of agricultural and food products to the total impacts of EU consumption on freshwater and marine eutrophication. Finally, grassland/pasture/meadow land occupation and arable land occupation are among the three flows most contributing to impacts on land use (respectively contributing to 31 and 21% of the total impact), while being mostly (62–72%) generated along the supply chain of agricultural and food products.

#### 3.2.3 Human toxicity (cancer and non-cancer) and ecotoxicity

**Fig. 3:**
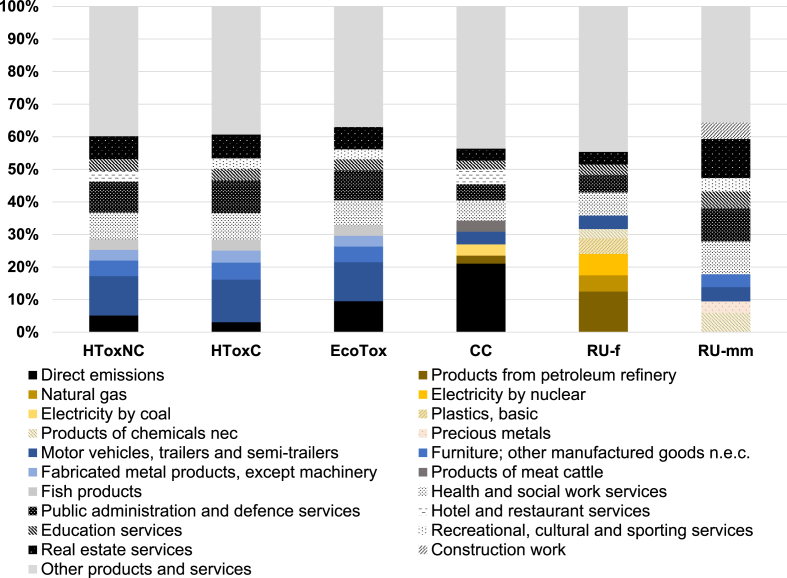
The ten categories of products and services (including direct emissions and use) most contributing to impacts of EU final consumption considering Human Toxicity, Non-Cancer (HToxNC), Human Toxicity, Cancer (HToxC), Ecotoxicity freshwater (EcoTox), Climate Change (CC), Resource Use, fossils (RU-f) and Resource Use, minerals and metals (RU-mm).

The ten categories of products and services (including direct emissions) most contributing to the impacts of EU final consumption are observed to be almost identical whether considering human toxicity, cancer, human toxicity, non-cancer and freshwater ecotoxicity, still with a slightly different ranking from one impact category to the other (Fig. 3). Firstly, several of the expenditures on services already observed as “key” to all impact categories are also part of the “Top 10” for these three impact categories. Expenditures relative to Real estate services, Public administration and defence services; compulsory social security services, Health and social work services, Education services and Recreational, cultural and sporting services contribute in total to 30–32% of the total impacts on human toxicity (cancer and non-cancer) and freshwater ecotoxicity. Moreover, beyond services, several (metal-based) manufactured products are also observed as relatively large contributors. In particular, Motor vehicles, trailers and semi-trailers stand for the most contributing category of products and services with respect to these three impact categories, standing for 12% to 13% of the total impacts. In the meantime, Furniture and other manufactured goods n.e.c. as well as Fabricated metal products, except machinery and equipment also rank in the Top 10, while several other metal-based manufactured products (e.g. Radio, television and communication equipment and apparatus) are among the 20 most contributing categories of products and services.

Impacts from EU consumption on human toxicity and ecotoxicity are almost exclusively induced by metal emissions to air, which overall stand for more than 99% of the total impacts. A limited set of emissions induces the majority of the impacts: chromium emissions to air contribute to 82% of the total impact on human toxicity, cancer, chromium and zinc emissions to air contribute to 83% of the impact on freshwater ecotoxicity, and mercury and lead emissions to air contribute to 88% of the total impact on human toxicity, non-cancer. Moreover, the profile of most contributing emissions is in many cases very similar irrespectively of the category of products and services. For example, considering 47 out of the 50 categories of products and services most contributing to the impacts of EU final consumption on human toxicity, cancer (including manufactured products, services, agricultural and food products, etc.), the contribution of chromium emissions to air ranges between 80 and 88%. A very similar feature is observed regarding impacts on non-cancer human toxicity and freshwater ecotoxicity: if considering any of the 50 most contributing categories of products and services, in most cases the shares of the corresponding most contributing substances are very similar to the ones observed with respect to the average EU final consumption.

#### 3.2.4 Photochemical ozone formation and particulate matter

Direct emissions in the consumption phase (mostly from households) stand for the main contribution to impacts on photochemical ozone formation and particulate matter (Table 1), reminding that this result is highly dependent on the version of EXIOBASE 3 implemented for calculations (with much lower contribution if considering direct emissions of PM_2.5_ and NMVOCs from final consumption from EXIOBASE 3.3.14). Emissions of PM_2.5_ to air stand for 88% of the impact on particulate matter, while emissions of NMVOCs and NO_x_ stand for respectively 63% and 27% of the impacts on photochemical ozone formation (Fig. 2). Here again, the implementation of EXIOBASE 3.3.14 to account for the direct emissions of PM_2.5_ and NMVOCs offers a different picture: one firstly observes a lower contribution of PM_2.5_ to particulate matter (55%) and on the contrary a larger contribution of NH_3_ and SO_x_ (respectively 26% and 15%), and secondly a lower contribution of NMVOCs to photochemical ozone formation (45%) and on the contrary a larger contribution of NO_x_ (41%).

#### 3.2.5 Climate change

The pattern of contributions to impact on climate change is specific, and any correspondence with other impact categories is observed to be only limited. Direct emissions from final consumption, mainly as CO_2_ and CH_4_, overall stand for 21% of the total impact of EU final consumption on climate change (Table 1 and Fig. 3). Considering the total impact of EU final consumption on climate change (that is, not only direct emissions but also including emissions along the supply-chain of products and services), the contributions of CO_2_, CH_4_ and N_2_O emissions to air respectively amount to 70%, 22% and 5% (Fig. 2).

Moreover, several of the expenditures on services already observed as “key” to all impact categories are also part of the 10 categories of products and services most contributing to impact on climate change. Expenditures relative to Health and social work services, Public administration and defence services; compulsory social security services, Hotels and restaurants services, Real estate services and Education services all together contribute to 22% of the total climate change footprint of EU consumption. Furthermore, several meat (in particular cattle and pigs), fish and dairy products, and more generally food products, are part of the 20 most contributing categories of products and services. Overall, the consumption of food products, beverages and tobacco products, as re-aggregated according to the NACE nomenclature, stands for 14% of the total impact on climate change, whereas representing 5% of the total final consumption expenditures (Eurostat, 2018). Similarly, Electricity respectively by coal and by gas appears within the 20 most contributing products and services with respect to climate change, so that the consumption of electricity overall stands for 6% of the impact on climate change.

#### 3.2.6 Fossils and minerals and metals resource uses

The contributions of products and services to impacts on resource use, respectively fossils and minerals and metals, shows on the one hand several similar features. Firstly, as is the case for most other impact categories, expenditures on services already observed as “key” to all impact categories are also part of the 10 categories of products and services most contributing to resource uses (Fig. 3). In the case of minerals and metals resource use, expenditures relative to Health and social work services, Public administration and defence services; compulsory social security services, Real estate services, Education services, Recreational, cultural and sporting services, as well as Construction work, are part of the “Top 10” and altogether contribute to 47% of the impact.

Moreover, in both cases of impact categories, raw materials and basic products show a relatively important contribution. In the case of fossils resource use, a relatively large contribution of fossil-based energy carriers is firstly observed. Products from petroleum refinery, electricity by nuclear and natural gas are respectively the 1st, 3rd and 5th contributors to impact. The re-aggregated contribution of electricity consumption amounts to 12% of the total impact on fossils resource use. Furthermore, but to a lower extent, fossil-based basic products (namely Plastics, basic and Products of chemicals nec) also show relatively large contributions.

Crude oil, natural gas and uranium are the three elementary flows representing the largest share in impact on fossils resource use, altogether contributing to 76% of the total impact of EU consumption (Fig. 3). Similarly, two elementary flows contribute to the majority of the impact on minerals and metals resource use: respectively other industrial minerals (contributing to 57% of the total impact) and gold (33%). On the one hand, at the inventory level, other industrial minerals represent 51% of the total mass of resources embodied in EU consumption (i.e. of the material footprint of EU consumption), that is twice larger than the share of sand and gravel in the total. On the other hand, at the impact assessment level, the CF for other industrial minerals has been set as a weighted average relative to the resource flows it is made of (SI document 2). It is driven by sulphur (the main globally produced other industrial mineral), resulting in a CF three orders of magnitude larger than that of iron, and five orders of magnitude larger than that of aluminium.

### 3.3 Contribution analysis by country of the EU28

**Fig. 4:**
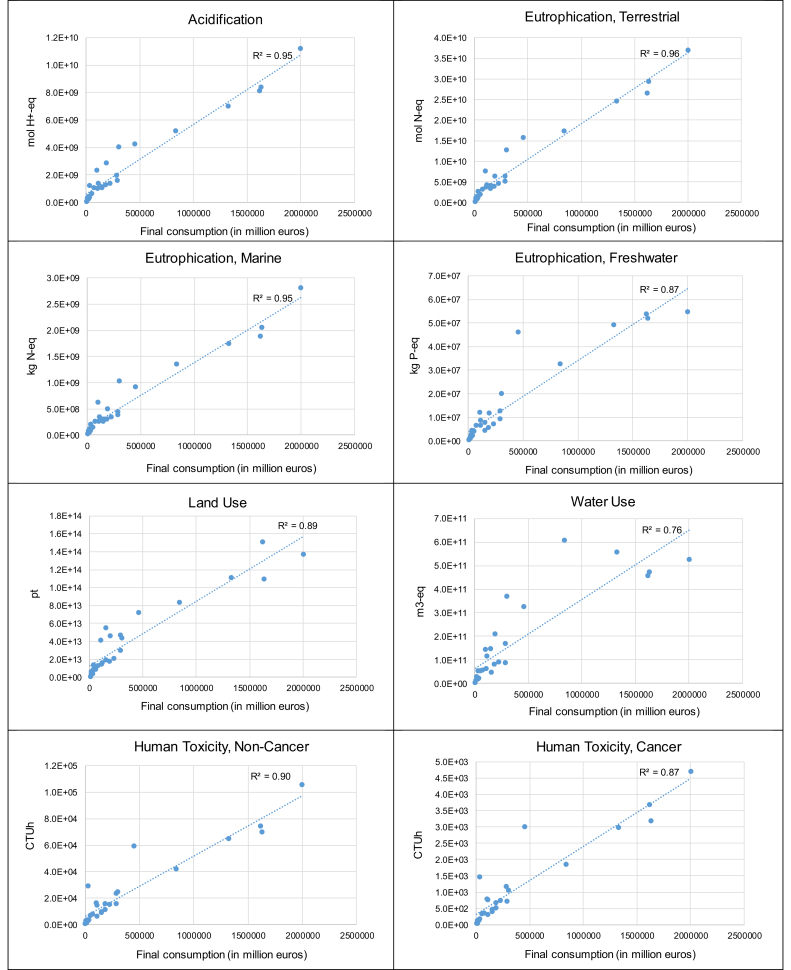

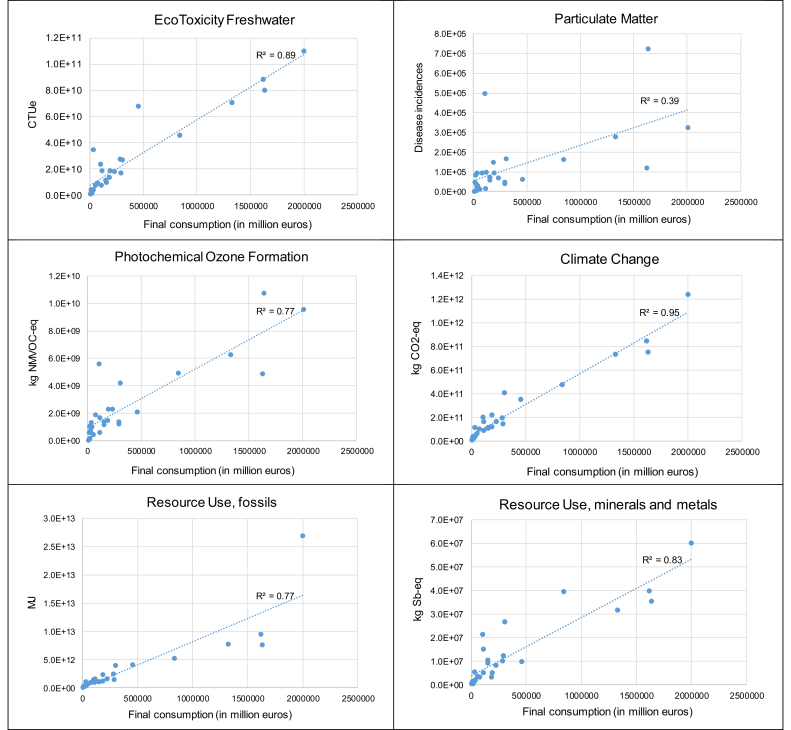
Environmental impacts of final consumption considering the 28 EU Member States and 14 impact categories, as a function of the total volume of final consumption expenditures.

**Fig. 5:**
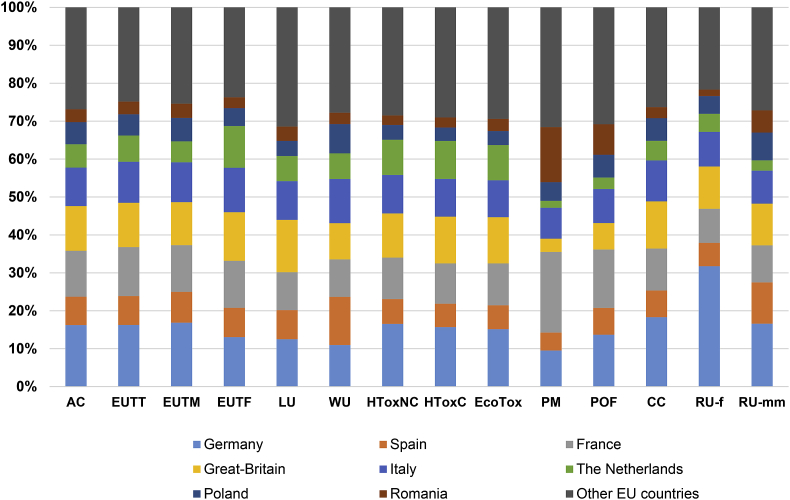
Contribution of EU Member States to the environmental impacts of EU final consumption.

The total volume of final consumption expenditures, in monetary terms, varies from one EU country to the other, with for example total consumption in Germany, France and Great Britain up to two orders of magnitude larger than that in Malta, Estonia, Latvia and Cyprus (SI document 1). The total volume of expenditures appears a key explanatory variable to the impacts induced by each Member State of the EU28 (Table 1 and Fig. 4). This is particularly true regarding climate change, eutrophication (terrestrial and marine) and acidification, for which the correlation coefficient (R^2^) between volume of expenditures and environmental impact is in the range [0.95–0.96]. Final consumption in Germany appears the most contributing to the environmental impacts of EU28 consumption, ranking first considering 10 impact categories out of the 14 under study (Fig. 5). In many cases of impact categories (in particular acidification, eutrophication, human toxicity, ecotoxicity and climate change), French and British final consumptions rank second and third.

However, regarding some impact categories, there is a discrepancy between the level of expenditures and the impact of final consumption. This is more particularly the case regarding particulate matter, for which the correlation coefficient (R^2^ = 0.39) is much lower than in any other case of impact categories. Similar observations (yet to a lower extent) can be made regarding water use, photochemical ozone formation and fossils resource use (R^2^ ∼ 0.76–0.77). In particular, whereas Spain is the 5th or 6th contributing country regarding almost all impact categories, it is the most contributing one regarding water use, with a share in the total impact (13%) that is approximately twice the one observed for most other impact categories. Yet, here again, the observation regarding particulate matter and photochemical ozone formation is highly dependent on the version of EXIOBASE 3 implemented for calculations. A good correlation is observed between volume of expenditures and environmental impact (R^2^ ∼ 0.92–0.93) if considering the direct emissions of PM_2.5_ and NMVOCs from final consumption from EXIOBASE 3.3.14 instead of EXIOBASE 3.3.8.

### 3.4 How far the environmental extensions of EXIOBASE 3 are suited for environmental impact assessment?

Environmental extensions of EXIOBASE 3 show several limitations that affect the robustness of the environmental impact assessment step. Firstly, a number of elementary flows are not reported in the environmental extensions, preventing to quantify part or even the entirety of environmental impacts with respect to several impact categories. As a first consequence, impacts on ozone depletion and ionizing radiations are required to be excluded from the impact assessment step using EXIOBASE 3, whereas they are recommended to be quantified in the EF2017 LCIA method. Similarly, neither emissions of pesticides and insecticides, nor emissions of metals to water are inventoried in EXIOBASE 3, while emissions of metals to soil are limited to zinc and lead emitted from the incineration sector. This implies underestimating the toxicity and ecotoxicity impacts of EU final consumption, to an extent that is unknown but still expected to be relatively significant in light of the contributions of chromium, zinc and folpet emissions to soil in terms of global impacts on human toxicity (non-cancer) and ecotoxicity (Crenna et al., 2019). As an additional consequence of the absence of several emissions of metals and pesticides in EXIOBASE environmental extensions, impacts of EU final consumption on human toxicity (cancer and non-cancer) and ecotoxicity are calculated to be almost entirely induced by emissions of metals to air, with limited differences in their share from one category of products and services to the other. It is expected, but yet should be further investigated, that the absence of part or of the entirety of pesticides, insecticides, and metal emissions in EXIOBASE may affect the intensity in human toxicity and freshwater ecotoxicity of many products along their supply-chain (e.g. agricultural and food products, metal-based fabricated products, etc.) and subsequently the contribution of these products to the total impacts of EU consumption.

A second limitation of EXIOBASE environmental extensions lies in the level of details of some elementary flows, which does not always match that of up-to-date impact assessment methods. In particular, in EXIOBASE 3, no specification is provided relatively to the emissions compartment features of PM_2.5_ to air (e.g. urban/rural, low/high stack), so that they are mapped as “unspecified” in the impact assessment phase. Within the EF2017 LCIA recommended method for particulate matter quantification, the CF for unspecified emissions of PM_2.5_ is set identical to that of PM_2.5_ emissions to urban air, close to ground. As a matter of comparison, one may note that, in the EF2017 LCIA method, the CF relative to unspecified emissions is accordingly set as respectively 21 and 7 times larger than the CFs relative to emissions to non-urban air close to ground, and to urban air from a very high stack. If considering, as a sensitivity analysis, that direct emissions from final consumption are emitted close to ground, either to urban air (75%) or to non-urban air (25%), while emissions from economic activities are emitted to urban air from a very high stack, then the total impacts of EU final consumption on particulate matter are quantified to be 28% lower than those calculated in the base case. Similarly, no speciation of metal emissions is reported in the environmental extensions of EXIOBASE 3. This is of particular importance regarding the quantification of impacts relative to chromium emissions to air, which contribute to 82% of the total impact on human toxicity (cancer) while classified as “unspecified” considering the framework of the EF2017 LCIA method. In the latter, the CF relative to unspecified chromium is set as the arithmetic mean of CFs relative to Cr VI and Cr III. As a sensitivity analysis highlighting the influence of this 50/50 assumption on impact assessment with EXIOBASE 3, one now considers that chromium VI represents 1/3 of chromium emissions whereas chromium III represents 2/3. The corresponding total impacts of EU final consumption on human toxicity, cancer are quantified to be 28% lower than in the base case.

Finally, a third issue is the level of aggregation of several elementary flows, reported at a coarse level compared to their counterparts in the impact assessment methods. This is more particularly the case with respect to minerals and metals resource use, for which several flows are highly aggregated (in particular, other metal ore and other industrial minerals). Their corresponding CFs, estimated as a weighted average considering annual global productions, are expected to encompass a relatively large uncertainty. In a context where the uncertainty in the CFs propagates to the impact assessment results, the large contribution of other industrial minerals to the total impact on minerals and metals resource use (57%) is consequently expected to entail in the meantime a large uncertainty in the total impact. Moreover, and similarly, the quantification of impact of EU consumption on land use currently only distinguishes the five elementary flows reported in EXIOBASE 3, at the highest level of land use classes (e.g. arable without any further specification). Once again, the level of details on flows in EXIOBASE 3 environmental extensions prevents to use entirely the full capacities of the impact assessment method, which provides characterization factors at a much finer level of disaggregation.

However, it should be reminded that, despite the uncertainties and limitations described above, EXIOBASE provides a life cycle inventory (LCI) with a level of completeness at 0% cut-off in terms of which flows in economy are accounted for. The extent to which this outweighs the lack of completeness in terms of number of included elementary flows in EXIOBASE compared to process-based LCI databases should be further explored. Moreover, compared to most LCI databases, the product classification in EXIOBASE is much more aggregated. Yet, the complete nature of EXIOBASE and consistent classification, based on acknowledged industry and product classification systems, enable for further subdividing industries and products to reach a higher granularity for contribution analysis in the future.

## 4 Conclusions and perspectives

This study has enabled to quantify the environmental impacts induced by final consumption in EU28, and accordingly to build a hierarchy of “key products and services” and “key substances” as a function of impact categories. The environmental impacts of EU consumption are mainly induced along the supply-chain of products and services as compared to the direct emissions from the consumption phase.

Food products, in particular meat and dairy products, are identified as key contributors regarding acidification, eutrophication, land use, water use, and climate change (still, to a lower extent regarding the latter). It is to be noted that the contribution of the whole food system, beyond food products only, is expected to be even larger. Indeed, the whole food system entails as well food consumed beyond outside household consumption, i.e. in canteens, schools, social care institutions and restaurants, or if accounting for the effect of indirect land use changes, which is excluded from this study while potentially influencing the impacts of food products on climate change. Besides, several manufactured products, raw materials and basic products importantly contribute to some impacts among human toxicity, freshwater ecotoxicity and resource uses. Moreover, several expenditures relating to services (e.g. Health and social work services) represent a significant share in the total final consumption of EU28 and at the same time a relatively large contribution to all impacts, despite a relatively low impact intensity. The impacts associated with these services include both direct and indirect (along the supply-chain) impacts, therefore accounting for impacts from e.g. food and electricity intermediate consumptions of these service sectors, that may play an important role (however, a decomposition analysis would be required to scrutinize this further). Overall, several distinct areas of consumption therefore drive distinct types of environmental impacts: implementing a multi-criteria approach appears key when assessing the environmental impacts of consumption as a support to policy-making.

As an IO database, EXIOBASE 3 enables to avoid any cut-off on economic flows in the environmental assessment of EU consumption, contrarily to process-based Life Cycle Assessment studies. It enables to account not only for products but also for services, both as final consumption and as intermediate consumption of economic activities, and accordingly provides the representation of the “true” entire footprint of EU consumption. Moreover, EXIOBASE 3 is based on a truly global inventory of product transactions and elementary flows. Finally, EXIOBASE 3 has been built considering a specific module for calculating and representing the environmental extensions of the agricultural sector, not only relying on statistical data but additionally combining process-based data at a relatively largely disaggregated level (Merciai and Schmidt, 2018). This specific feature of EXIOBASE 3 is key in the evaluation of the environmental impacts of EU final consumption, in a context where food products show a significant contribution to impacts on acidification, eutrophication, land use and water use along their supply-chain.

However, at this stage of development of the environmental extensions of EXIOBASE 3, three of their features imply that the impact assessment step adds a layer of uncertainty, potentially significant but still unexplored:

- a number of elementary flows are absent from the environmental extensions, so that part (especially regarding human toxicity and ecotoxicity) or even the entirety of impacts (regarding ozone depletion and ionizing radiations) cannot be properly assessed;
- details are missing regarding some properties of emissions (e.g. regarding the specificity of the emission compartment in the case of PM_2.5_ or the oxidation state of chromium emissions ), whereas they may significantly affect the impact assessment step;
- some flows are reported in a very aggregated manner compared to their counterpart in impact assessment methods, while largely contributing to impacts (e.g. other industrial minerals with respect to minerals and metals resource use).

These three features could be considered a basis for completing in future the environmental extensions in order to improve the robustness of any impact assessment performed using EXIOBASE 3, specifically regarding human toxicity (cancer and non-cancer), freshwater ecotoxicity, minerals and metals resources and to a lower extent particulate matter and land use. Any conclusion drawn regarding these six impact categories, in this study or in any study that undertakes a similar approach of impact assessment (e.g. Steinmann et al., 2018), should be considered keeping in mind the potential uncertainty encompassed in impact assessment results.
