# Supplementary material for: Assessing the environmental impacts of EU consumption at macro-scale
Source: J Clean Prod. 2019 Apr 10;216:382–93. doi: 10.1016/j.jclepro.2019.01.134 (PMC6472615; doi:10.1016/j.jclepro.2019.01.134)
Supplement: Multimedia component 1 [file mmc1.pdf]

Supporting Information document 2

-

Assessing the environmental impacts of EU consumption at macro-scale

Antoine Beylot[1], Michela Secchi[1], Alessandro Cerutti[1], Stefano Merciai[2], Jannick Schmidt[3], Serenella Sala[1*]

1: European Commission-Joint Research Centre, Via Enrico Fermi 2749, I-21027 Ispra (VA), Italy

2: 2.-0 LCA consultants, Rendsburggade 14, room 1.431, 9000 Aalborg, Denmark

3: Danish Center for Environmental Assessment (DCEA), Department of Planning, Aalborg University.

Rendsburggade 14, room 1.431, 9000 Aalborg, Denmark

* Corresponding author: serenella.sala@ec.europa.eu

Telephone: +39 0332 786417

Contents

Using the hybrid version of EXIOBASE 3 to assess the environmental impacts of consumption: a practical guide 2

Table A1: Coverage of elementary flows in the 16 impact categories of the Environmental Footprint (EF) 2017 LCIA method (EC, 2017) 4

Table A2: Elementary flows of EXIOBASE 3 environmental extensions assigned with a characterization factor in this study, based on the EF2017 LCIA method 5

Figure A1: Environmental impacts by type of final consumption expenditures, calculated using EXIOBASE 3 and considering 14 impact categories, compared with the share of expenses of each type of final consumption (Eurostat, 2018) 6

Table A3: Environmental impacts of EU final consumption in 2011: the nine categories of products and services being part of the "Top 20 contributors" with respect to the 14 impact categories under study, and their ranking among the 137 categories of products and services consumed 7

Figure A2: Impact intensity of 1 product and 3 service categories identified as "key" for all impact categories under study (in impact unit per Euro), compared with the average impact intensity of EU28 final consumption in 2011 (base 100% for the average impact intensity) 8

Figure A3: Total final consumption expenditures per EU Member State, in 2011 (Eurostat, 2018) 9

Figure A4: Total final consumption expenditures per EU Member State in 2011, expressed in % of the GDP (Eurostat, 2018) 10

References 11

Using the hybrid version of EXIOBASE 3 to assess the environmental impacts of consumption: a practical guide

This section describes how to use the hybrid version of EXIOBASE 3 to assess the environmental impacts of consumption. This approach has been applied specifically considering European Union (EU) final consumption (that is, the sum of expenditures from households, from government and from Non-Profit Institutions Serving Households). A similar approach could for example be applied to a share of EU final consumption (e.g. only considering household consumption), or to the consumption or trade exchanges of any country or region distinguished in EXIOBASE 3.

Where to access the hybrid version of EXIOBASE 3?

The hybrid version of EXIOBASE 3 can be freely accessed and downloaded from the following link:

https://www.exiobase.eu/index.php/data-download/exiobase3hyb (last visit 20/11/2018)

What does EXIOBASE 3 contain? Which are the files for use in the calculations?

As mentioned in the article, the inventory (*g*) of emissions to the environment, and of resources extracted from the environment, as a response to a given final demand is calculated according to:

$g = Bx = B(I - A)^{-1}f$ (equation 3 in the article)

Any user of EXIOBASE 3 can implement this calculation by use of a computing environment such as R, MatLab or similar, considering the data downloaded from the above-mentioned link:

- *f* stands for the final demand. In the downloaded database, *f* is reported in two forms: as an Excel document (“Exiobase_MR_HFD_2011_v3_3”) and as a text document (“Y”). In this study, only the final demand associated with the 28 EU Member States has been considered in the calculations;
- *B* stands for the matrix of sectorial resources and emissions intensities. In the downloaded database, *B* is reported as a text document (entitled “F”). Alternatively, *B* can be built by users in the form of an Excel document (considering the two Excel documents: “MR_HSUT_2011_v3_3_extensions” and “Exiobase_MR_HIOT_2011_v3_3_by_prod_tech”, worksheet “Principal_production_vector”);
- *A* stands for the technological requirement matrix. In the downloaded database, *A* is reported as a text document (entitled “A”). Alternatively, matrix *A* can be built by users in the form of an Excel document, considering the two worksheets “HIOT” and “Principal_production_vector” of the Excel document “Exiobase_MR_HIOT_2011_v3_3_by_prod_tech”;
- Finally, *I* is the identity matrix.

How to calculate the environmental impacts of EU consumption?

As described in the article, once the vector of environmental pressures induced by EU final consumption is obtained (*g*, as calculated from equation 3), the corresponding impacts are calculated according to:

$e = Cg$ (equation 5 in the article)

with *e* the vector of environmental impacts and *C* the matrix of characterization factors (reporting the impact intensity per unit of resource extracted or substance emitted to the environment). Matrix *C* has been specifically constructed in this study (see in particular Supporting Information document 1 for details on the construction), under the form of an Excel document, considering one worksheet for each impact category under study (14 in total). The calculations described in equation 5 have been performed using Excel, but could have been performed as well considering other computing environments (such as e.g. the LCA software SimaPro, or R).

Table A1: Coverage of elementary flows in the 16 impact categories of the Environmental Footprint (EF) 2017 LCIA method (EC, 2017)

	**Number of substances**						
	**Total**	**By compartment**					
Acidification	9	9	to air only				
Climate change	212	212	to air only				
Ecotoxicity, freshwater	7566	2524	to air	2521	to soil	2521	to water
Eutrophication, freshwater	8			4	to soil	4	to water
Eutrophication, marine	11	6	to air			5	to water
Eutrophication, terrestrial	7	7	to air only				
Ionizing radiation	42	21	to air			21	to water
Human toxicity, non-cancer	1321	443	to air	439	to soil	439	to water
Human toxicity, cancer	1816	606	to air	605	to soil	605	to water
Ozone depletion	26	26	to air only				
Particulate matter	11	11	to air only				
Photochemical ozone formation	135	135	to air only				
Resource use, fossils	6						
Resource use, minerals and metals	48						
Water use	7	all with the same CF					
Land use	57	occupation					

Table A2: Elementary flows of EXIOBASE 3 environmental extensions assigned with a characterization factor in this study, based on the EF2017 LCIA method

	Number of substances						
	**Total**	**By compartment**					
Acidification	3	3	to air only				
Climate change	4	4	to air only				
Ecotoxicity, freshwater	15	13	to air	2	to soil	0	to water
Eutrophication, freshwater	2	0	to air	1	to soil	1	to water
Eutrophication, marine	3	2	to air	0	to soil	1	to water
Eutrophication, terrestrial	2	2	to air only				
Ionizing radiation	Not considered in this study, as there is no emission relative to ionizing radiation in EXIOBASE 3 environmental extensions						
Human toxicity, non-cancer	11	9	to air	2	to soil	0	to water
Human toxicity, cancer	11	10	to air	1	to soil	0	to water
Ozone depletion	Not considered in this study, due to the incompleteness of EXIOBASE 3 environmental extensions regarding ozone-depleting substances						
Particulate matter	4	4	to air only				
Photochemical ozone formation	5	5	to air only				
Resource use, fossils	9						
Resource use, minerals and metals	13						
Water use	1						
Land use	5	occupation					

Figure A1: Environmental impacts by type of final consumption expenditures, calculated using EXIOBASE 3 and considering 14 impact categories, compared with the share of expenses of each type of final consumption (Eurostat, 2018)

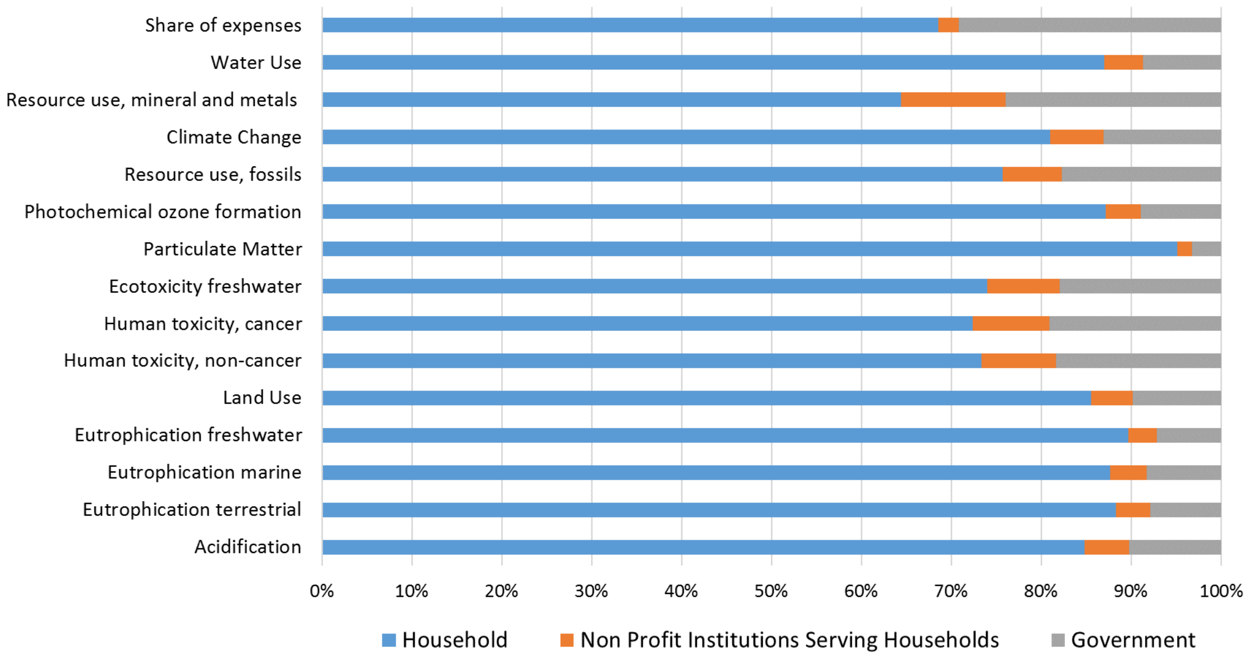

Table A3: Environmental impacts of EU final consumption in 2011: the nine categories of products and services being part of the “Top 20 contributors” with respect to the 14 impact categories under study, and their ranking among the 137 categories of products and services consumed

Note for reading this table: e.g. “1” in this table indicates that the corresponding product or service is the most contributing one among the 137 consumed

	AC	EUTT	EUTM	EUTF	LU	WU	HToxNC	HToxC	EcoTox	PM	POF	CC	RU-f	RU-mm
**Food products nec**	9	8	7	9	9	2	16	15	15	10	16	14	16	14
**Products of chemicals nec**	14	18	19	17	19	19	13	16	16	12	10	12	10	4
**Motor vehicles, trailers and semi-trailers**	7	15	14	19	17	15	1	1	1	5	4	4	7	7
**Hotels and restaurants services**	2	3	1	2	2	1	8	10	10	2	3	3	12	11
**Real estate services**	10	13	13	15	6	16	4	4	4	7	5	5	8	1
**Public administration and defence services; compulsory social security services**	8	10	9	12	7	11	2	2	2	4	2	2	4	2
**Education services**	12	11	11	13	13	14	6	7	6	9	12	8	9	5
**Health and social work services**	5	6	6	6	3	4	3	3	3	1	1	1	2	3
**Recreational, cultural and sporting services**	17	16	16	18	18	20	10	9	9	13	14	11	13	8

Note on abbreviations: AC stands for Acidification; EUTT for Eutrophication Terrestrial; EUTM for Eutrophication Marine; EUTF for Eutrophication Freshwater; LU for Land Use; WU for Water Use; HToxNC for Human Toxicity, Non-Cancer; HToxC for Human Toxicity, Cancer; EcoTox for Ecotoxicity freshwater; PM for Particulate Matter; POF for Photochemical Ozone Formation; CC for Climate Change; RU-f for Resource Use, fossils; RU-mm for Resource Use, minerals and metals.

nec : not elsewhere classified

Figure A2: Impact intensity of 1 product and 3 service categories identified as “key” for all impact categories under study (in impact unit per Euro), compared with the average impact intensity of EU28 final consumption in 2011 (base 100% for the average impact intensity)

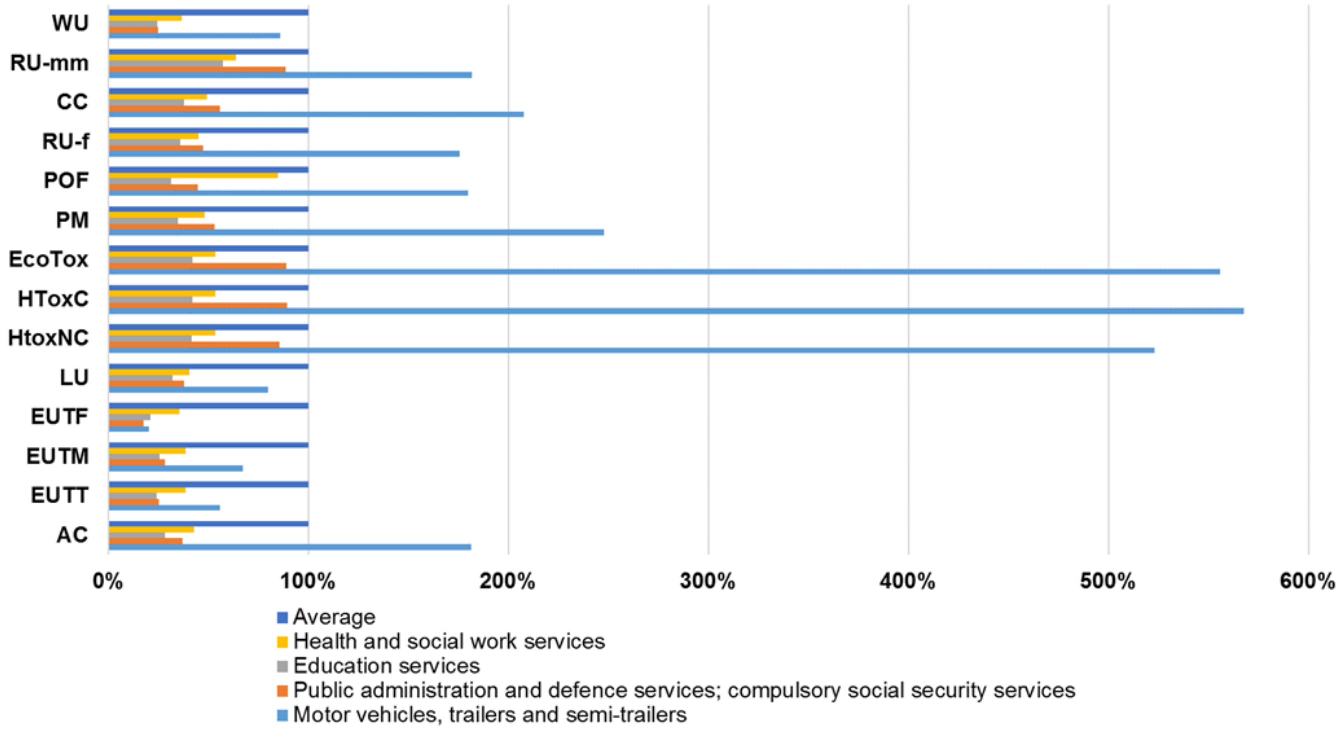

Note on abbreviations: AC stands for Acidification; EUTT for Eutrophication Terrestrial; EUTM for Eutrophication Marine; EUTF for Eutrophication Freshwater; LU for Land Use; WU for Water Use; HToxNC for Human Toxicity, Non-Cancer; HToxC for Human Toxicity, Cancer; EcoTox for Ecotoxicity freshwater; PM for Particulate Matter; POF for Photochemical Ozone Formation; CC for Climate Change; RU-f for Resource Use, fossils; RU-mm for Resource Use, minerals and metals.

Figure A3: Total final consumption expenditures per EU Member State, in 2011 (Eurostat, 2018)

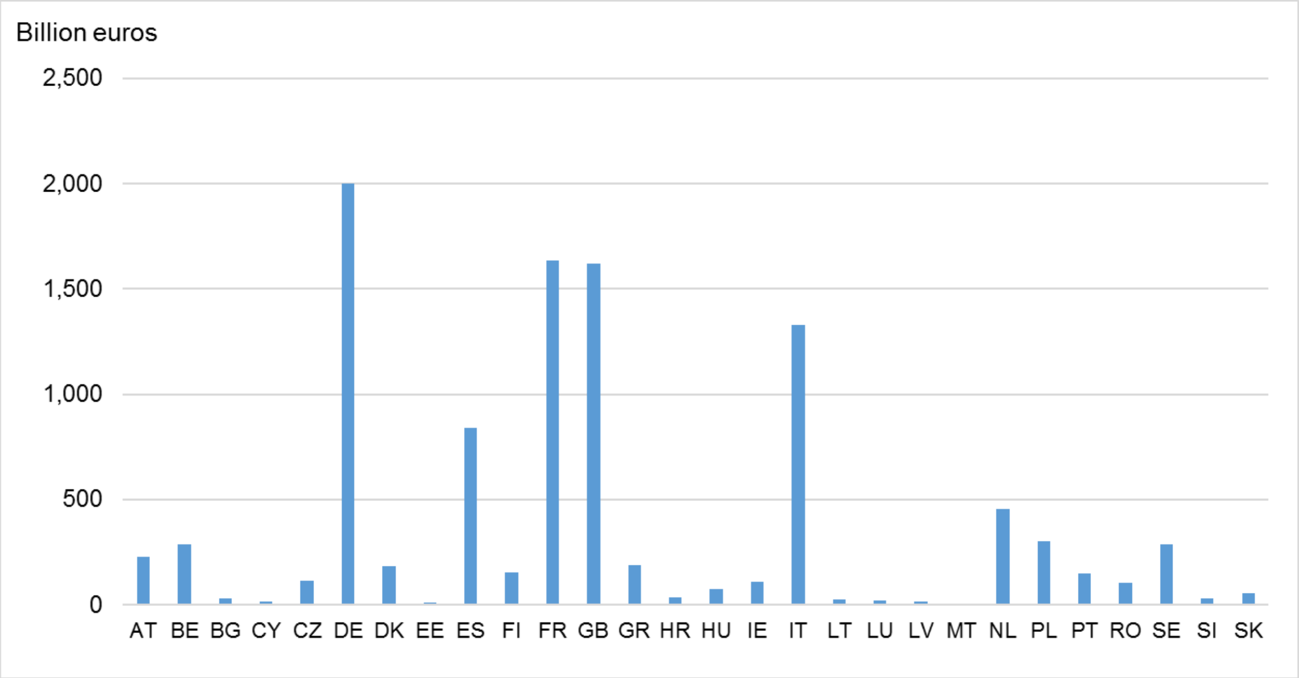

AT Austria; BE Belgium; BG Bulgaria; CY Cyprus; CZ Czech Republic; DE Germany; DK Denmark; EE Estonia; ES Spain; FI Finland; FR France; GB United Kingdom; GR Greece; HR Croatia; HU Hungary; IE Ireland; IT Italy; LT Lithuania; LU Luxemburg; LV Latvia; MT Malta; NL The Netherlands; PL Poland; PT Portugal; RO Romania; SE Sweden; SI Slovenia; SK Slovakia.

Figure A4: Total final consumption expenditures per EU Member State in 2011, expressed in % of the GDP (Eurostat, 2018)

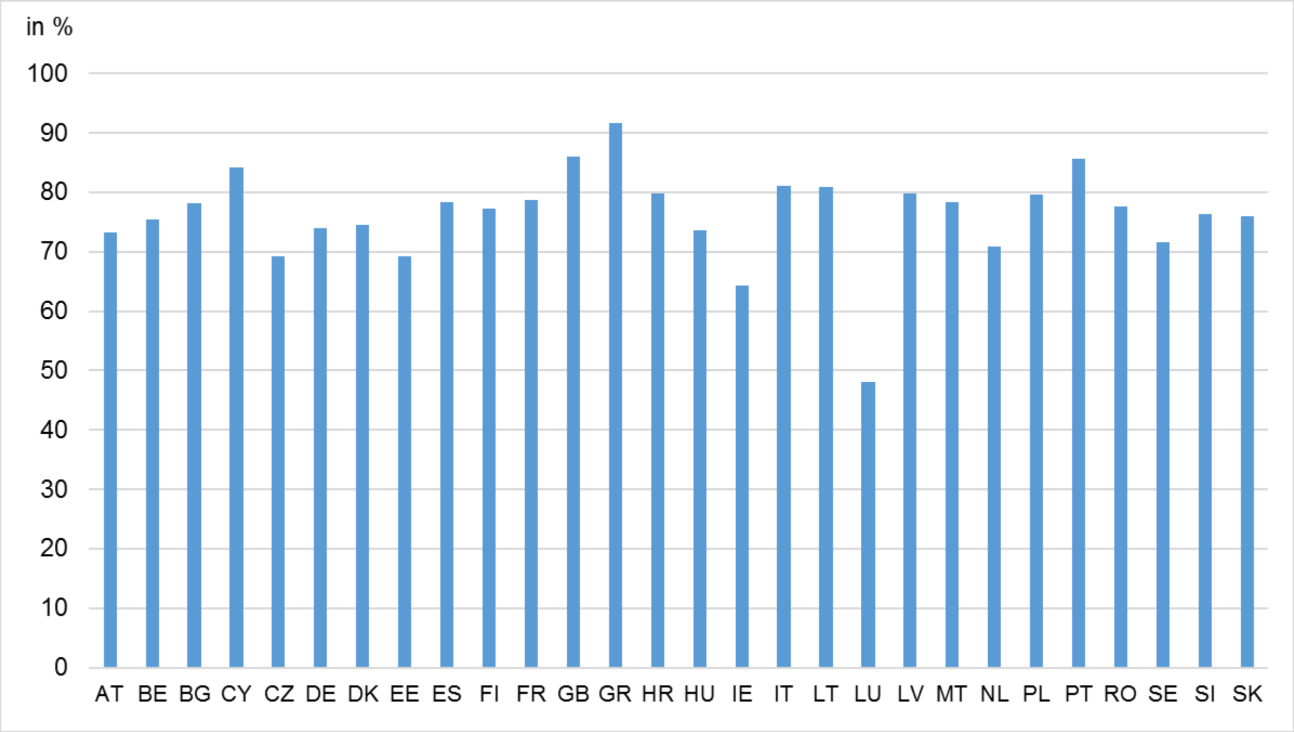

AT Austria; BE Belgium; BG Bulgaria; CY Cyprus; CZ Czech Republic; DE Germany; DK Denmark; EE Estonia; ES Spain; FI Finland; FR France; GB United Kingdom; GR Greece; HR Croatia; HU Hungary; IE Ireland; IT Italy; LT Lithuania; LU Luxemburg; LV Latvia; MT Malta; NL The Netherlands; PL Poland; PT Portugal; RO Romania; SE Sweden; SI Slovenia; SK Slovakia.

References

EC, 2017. PEFCR Guidance document - Guidance for the development of Product Environmental Footprint Category Rules (PEFCRs), version 6.3, December 2017. Available at: http://ec.europa.eu/environment/eussd/smgp/pdf/PEFCR_guidance_v6.3.pdf (Accessed July 2018)

Eurostat, 2018. Database. http://ec.europa.eu/eurostat (Accessed March 2018)
